# Corporate Social Responsibility and Access to Policy Élites: An Analysis of Tobacco Industry Documents

**DOI:** 10.1371/journal.pmed.1001076

**Published:** 2011-08-23

**Authors:** Gary J. Fooks, Anna B. Gilmore, Katherine E. Smith, Jeff Collin, Chris Holden, Kelley Lee

**Affiliations:** 1 School for Health, University of Bath, Bath, United Kingdom; 2 Centre for International Public Health Policy, University of Edinburgh, Edinburgh, United Kingdom; 3 London School of Hygiene and Tropical Medicine, London, United Kingdom; 4 Department of Social Policy and Social Work, University of York, York, United Kingdom; University of Queensland, Australia

## Abstract

**Background:**

Recent attempts by large tobacco companies to represent themselves as socially responsible have been widely dismissed as image management. Existing research supports such claims by pointing to the failings and misleading nature of corporate social responsibility (CSR) initiatives. However, few studies have focused in depth on what tobacco companies hoped to achieve through CSR or reflected on the extent to which these ambitions have been realised.

**Methods and Findings:**

Iterative searching relating to CSR strategies was undertaken of internal British American Tobacco (BAT) documents, released through litigation in the US. Relevant documents (764) were indexed and qualitatively analysed. In the past decade, BAT has actively developed a wide-ranging CSR programme. Company documents indicate that one of the key aims of this programme was to help the company secure access to policymakers and, thereby, increase the company's chances of influencing policy decisions. Taking the UK as a case study, this paper demonstrates the way in which CSR can be used to renew and maintain dialogue with policymakers, even in ostensibly unreceptive political contexts. In practice, the impact of this political use of CSR is likely to be context specific; depending on factors such as policy élites' understanding of the credibility of companies as a reliable source of information.

**Conclusions:**

The findings suggest that tobacco company CSR strategies can enable access to and dialogue with policymakers and provide opportunities for issue definition. CSR should therefore be seen as a form of corporate political activity. This underlines the need for broad implementation of Article 5.3 of the Framework Convention on Tobacco Control. Measures are needed to ensure transparency of interactions between all parts of government and the tobacco industry and for policy makers to be made more aware of what companies hope to achieve through CSR.

*Please see later in the article for the Editors' Summary*

## Abstract 2

Gary Fooks and colleagues undertook a review of tobacco industry documents and show that policies on corporate social responsibility can enable access to and dialogue with policymakers at the highest level.

## Editors' Summary

**Background:**

In the past, companies and multinational corporations were judged on the profits they made. Nowadays, though, much is made of corporate social responsibility (CSR). CSR is the commitment by business to behave ethically and to contribute to economic development while improving the quality of life of the workforce, their families, the local community, and society at large. Put simply, companies and corporations now endeavor to show that they have a positive impact on the environment, consumers, employees, and society in addition to making money for their shareholders. Large tobacco companies are no exception. British American Tobacco (BAT, the world's second largest publicly traded tobacco company), for example, began working on a wide-ranging CSR program more than a decade ago. Given that tobacco is responsible for an estimated 5.4 million deaths worldwide annually, this program was initially met with hostility and dismissed as an image management exercise. However, large parts of the investment and CSR communities now approve of BAT's CSR program, which has won numerous awards.

**Why Was This Study Done?:**

But what do BAT and other tobacco companies actually hope to achieve through their CSR initiatives and how successful have they been in achieving these aims? Few studies have addressed these important questions. In particular, there has been little research into the extent to which tobacco companies use CSR initiatives as a form of corporate political activity that can help them gain “access” to policymakers and define the legitimate concerns and optimal alternatives of public policy (“issue definition”). Access is defined as taking place when policymakers consider the views of policy advocates such as tobacco company employees and is a crucial component of issue definition, which refers to the strategies adopted by bodies such as multinational corporations to influence the policy agenda by defining what issues public policy should concern itself with and how it should approach them. In this case study, the researchers explore whether BAT's CSR program works as a form of corporate political activity by systematically examining internal BAT documents made publicly available as a result of US litigation. Specifically, the researchers examine BAT's efforts through its CSR program to reestablish access with the UK Department of Health following the department's decision in the late 1990s to restrict contact with major tobacco companies.

**What Did the Researchers Do and Find?:**

Using iterative searching, the researchers identified 764 documents in the Legacy Tobacco Documents Library (a large collection of internal tobacco company documents released as a result of US litigation cases) that contain information relevant to BAT's CSR strategies. Their analysis of these documents indicates that one of the key aims of the CSR program actively developed over the past decade by BAT was to help secure access to policymakers and shows how BAT used CSR to renew and maintain dialogue with policymakers at a time when contact between government and tobacco companies was extremely restricted. The documents also show that BAT employees used CSR initiatives as a means of issue definition to both optimize the probability of subsequent discussions taking place and to frame their content. Finally, the documents illustrate how BAT used its CSR program to expand the number of access points across government, thereby providing BAT with more opportunities to meet and talk to officials.

**What Do These Findings Mean?:**

These findings suggest that CSR is a form of corporate political activity that potentially has important implications for public health given the documented impact of the political activity of tobacco companies in delaying and blocking health-related tobacco control policies. In practice, the impact of the political use of CSR is likely to be context specific and will depend on factors such as whether senior policymakers regard companies as reliable sources of information. Importantly, these findings underline the need for broad implementation of Article 5.3 of the World Health Organization's Framework Convention on Tobacco Control (FCTC), an international treaty that calls for the introduction of multiple measures to reduce tobacco consumption, including tobacco advertizing bans and relevant taxation policies. Article 5.3 aims to protect public-health policies on tobacco control from tobacco industry influence. The findings of this study indicate that implementation of Article 5.3 will require measures that ensure transparency in interactions between all parts of government and the tobacco industry and will need an increased awareness across government of what tobacco companies hope to achieve through CSR.

**Additional Information:**

Please access these Web sites via the online version of this summary at http://dx.doi.org/10.1371/journal.pmed.1001076.

- The Corporate Responsibility (CORE) coalition, an alliance of voluntary organizations, trade unions, and companies, maintains a Web site that contains useful material on corporate social responsibility
- The European Coalition for Corporate Justice (ECCJ) promotes corporate accountability by bringing together national platforms of civil society organizations (including NGOs, trade unions, consumer advocacy groups, and academic institutions) from all over Europe
- The Legacy Tobacco Documents Library is a public, searchable database of tobacco company internal documents detailing their advertising, manufacturing, marketing, sales, and scientific activities
- The World Health Organization provides information about the dangers of tobacco (in several languages), details of the Framework Convention on Tobacco Control (in several languages), and guidelines for the implementation of Article 5.3 of the FCTC
- The Framework Convention Alliance provides more information about the FCTC
- For information about tobacco industry influence on policy, see the 2009 World Health Organization report Tobacco interference with tobacco control

## Introduction

On December 3rd 2000, British American Tobacco (BAT) gave arguably the clearest indication yet of its decision to join the growing corporate social responsibility (CSR) movement [1] with the highly symbolic announcement of a £3.8 million donation to create a Centre for CSR at the University of Nottingham [2],[3]. Notice of BAT's endowment heralded the existence of a broader strategy that BAT had been working on since the spring of 1997 [4]. Unsurprisingly, news that the world's second largest publicly traded tobacco company—selling a product which is currently estimated to cause 5.4 million deaths a year worldwide [5]—was attempting to associate itself with the idea of CSR was initially met with a mixture of cynicism and hostility [6]–[10]. In the years following the announcement, however, BAT's CSR programme (CSRP) has been steadily accepted and approved by large parts of the investment and CSR communities. Senior employees are now frequently invited to speak at public engagements on CSR and business ethics [11]–[16] and the company has won numerous awards for its CSRP (see Box 1) [17].

**Box 1. Selective summary of awards for BAT's social and environmental programmes/reporting [137]**

2009:

- Received a Platinum rating in the UK Business in the Community Corporate Responsibility Index
- Selected for the 8th successive year as the only tobacco business in the 2009 Dow Jones Sustainability World Index (DJSI World) and the Dow Jones STOXX Sustainability Index (DJSI STOXX) for Europe, scored on economic, social, and environmental performance.

2008:

- Awarded a Gold ranking in the “Companies that Count 2008” list of the UK's 100 most responsible companies, published in the Sunday Times and based on the Business in the Community Corporate Responsibility Index.

2007:

- Ranked third out of the FTSE100 companies in the Ethical Bonus Index 2007 (compiled by intelligent Giving on and focusing on how companies enable staff to donate to charity, including allowing time off for volunteering, matching donations, and running a “payroll giving” service).
- Earned an overall score of 98.6% in the Business in the Environment Index run by Business in the Community (which placed the company in the highest possible Platinum performance band).

2006:

- Ranked in the Premier League of Business in the Community's Environment Index, as one of the 23 companies out of 155 participants to score over 95%
- Ranked joint 31st of the top 100 companies in Business in the Community's wider Corporate Responsibility Index (including “outstanding” scores for environmental management and marketplace management)

2004:

- Ranked fourth by the UN Environment Programme (UNEP) among 50 corporate social reporters (UNEP found the company's reporting on human rights and community development to be “world class” and gave the Group a score of 64% for its ethical, social, and environmental performance against an average score of 47%).

Whilst the above may represent relatively limited measures of the extent to which BAT has been accepted as a socially responsible corporation, the gradual acceptance of the company's CSRP is significant given that it has occurred despite widely available studies pointing to the existence of basic inconsistencies between many claims in its social reports and how it conducts its business in practice [18]–[25]. One reason for this acceptance might reside in the focus of the existing literature, which, arguably, does not pay sufficient attention to the essentially political nature of BAT's CSRP. Analysis of tobacco companies' motivation to develop CSR initiatives tends to focus on its efforts to restore legitimacy and manage reputation. Where studies do concern themselves with what we might broadly describe as political aspects of CSR, such as its use as a tool of regulatory management [18],[21],[25], these effects tend to be stated, rather than scrutinised and explained. As a result, there is currently a lack of depth in our understanding of what exactly BAT (and, to a lesser extent, other tobacco companies) hopes to gain from CSR, how its senior managers believe it might facilitate these ambitions, and how successful such strategies appear to have been.

This paper aims to build on the existing literature on CSR [26]–[28] by exploring how BAT's CSRP works as a form of corporate political activity. In keeping with our interest in undertaking a relatively detailed exploration of the practices and strategies that CSR makes available to large transnational companies like BAT, we have taken a case study approach to illustrate the relationship between CSR, access to political actors, and issue definition. Specifically our case study concerns BAT's efforts to reestablish access with the UK Department of Health (DoH), following the latter's decision to restrict contact with major tobacco companies. Although the focused nature of the case study means that we do not closely analyse CSR's other political effects such as constituency building and agenda setting, the DoH's decision does allow us to track the thinking behind, form, and relative success of different CSR-based techniques. Moreover, despite the geographical focus of the case study on the UK, the international presence of BAT and its promotion of CSR across its subsidiaries suggest the existence of similar practices in other countries in which BAT operates.

We define access as taking place when officials give consideration to the views of policy advocates (in this case BAT) [29]. This is commonly evidenced by meetings with company representatives. Access to policymakers is often a necessary precondition for achieving political influence [30]–[34]; indeed, political scientists often conceive it as an indicator of political influence [35] and a crucial component of agenda setting [36]. Issue definition (which is sometimes used interchangeably with agenda setting [37]) is typically used to refer to the strategies adopted by social actors (in this case, large multinational corporations) to define the legitimate concerns, appropriate reach, and optimal alternatives of public policy. In the present study, we primarily use the concept to draw attention to the way in which BAT used its CSRP in dialogue with policymakers in its efforts to influence the priorities of public and elected officials in the UK, encourage them to take notice of alternative modes of (voluntary) regulation being proposed by the company, and to revise their concerns about whether the industry could be trusted to work in partnership.

We propose that our case study underlines the value of understanding BAT's CSRP as an innovative form of corporate political activity [38]–[41]. This approach to conceptualising CSR has potentially important implications for public health given the widely documented impact of tobacco companies' political activity in delaying and blocking health related policies [42]–[44]. More generally, it is likely to be relevant to understanding the impact of CSR in other industrial sectors, such as alcohol and food, where CSR also seems to have been used to shape government policy [45],[46].

## Methods

The analysis is based on BAT documents made publicly available as a result of litigation in the US, and which are now available online at the Legacy Tobacco Documents Library (http://legacy.library.ucsf.edu/index.html). The case study emerged from a larger programme of work that aimed to explore the rationale, extent, and impact of BAT's CSR activities. A snowball approach was taken to searching the archive between April 2008 and March 2009. Initial searches used broad terms such as social responsibility, social reporting, and CSR. During this phase of our documentary searching, the issue of access emerged as an important potential line of enquiry. Further search terms were then selected to reflect the names of specific initiatives used to secure access, such as Partnership for Change, key BAT employees involved in developing BAT's CSRP and liaising with policymakers, and public and elected officials with whom BAT employees attempted to meet. In total 185 search terms have been used to retrieve 9,603 documents (many of which were duplicates) relating to CSR and social reporting. For the current study 764 documents, with a date range between 1998 and 2000, have been studied in detail and indexed. Analysis was based on an approach to company document analysis summarised by Forster and complemented by archival techniques recommended by Hill [47],[48]. Secondary data were obtained from newspaper reports and contacts in the field.

## Results

### Political Background

A brief understanding of the political context to our case study is crucial to understanding how BAT came to conceive of CSR as both a mode of securing access and as a way of reshaping the thinking of policymakers in the UK about how best to respond to the problem of widespread tobacco use. As a large multinational company registered in the UK, BAT was, historically, treated as a political insider by public and elected officials [49],[50]. This meant that it enjoyed privileged access to policymakers and was regularly consulted on plans for new government policy [51],[52]. The company relied on this close relationship in a number of different ways. Summarising an agreement by the UK Department of Trade and Industry (DTI) to act as the company's sponsor, a note from the company's External Affairs Manager records that the department was “particularly keen to help [BAT] against [its] foreign competitors” [53]–[55]. Other evidence indicates that the DTI was open to helping BAT break into new markets [56] and, until Labour's 1997 election victory, BAT had often relied on the UK to work with other member states to veto proposed EU tobacco control legislation [57],[58].

By the late 1990s, however, senior BAT managers had become concerned that its deteriorating relationship with the DoH was potentially symptomatic of a broader decline in its status as a political insider [38]. This feeling was underlined by the development of the Framework Convention on Tobacco Control (FCTC) [59]—an international treaty negotiated under the auspices of the World Health Organization (WHO)—and a series of planned tobacco control measures by the European Union (EU), which included proposals to curb tobacco advertising and tighten product regulation [57],[60],[61]. In the UK, these concerns had been intensified by the Labour Party's 1997 electoral success. In contrast to the Conservative Party, whose preference for voluntary forms of tobacco control [52] reflected its long-term ideological attachment to limited state intervention in the economy, Labour was prepared to push through domestic legislation and support proposals for new regulation in the EU [62]. In January 1998, the Labour Government published a white paper, “Smoking Kills,” that outlined proposals to abolish tobacco advertising and promotion, prevent tobacco smuggling, and proposed action on clean indoor air [63]. The following year the House of Commons Health Committee undertook a widely reported enquiry into the tobacco industry [64], and in 2000, the DTI launched a potentially damaging investigation into BAT's involvement in cigarette smuggling (see Box 2) [65]–[68]. Significantly, both of these were precipitated in part by the release of internal company documents [69], which, by deepening distrust of the industry amongst a range of political actors, was felt to have reduced the company's access to officials involved in policy discussions relevant to its business [70]–[74].

**Box 2. DTI investigation into BAT**

In the event, the DTI's investigation resulted in no further action being taken against BAT. The circumstances leading up to the decision was the subject of an investigation by the *Guardian* newspaper in 2004 [67]. Evidence of BAT's alleged complicity in smuggling had initially been presented to the House of Commons Health Committee inquiry whose report specifically called on the DTI to investigate BAT [64]. Given the serious and complex nature of the allegations the DTI was reported to be considering an inquiry under section 432 of the Companies Act 1985 [67], which gives DTI inspectors wide-ranging powers to seize files, summon witnesses, question them under oath, and require them to give every assistance in connection with the investigation. Further, inspectors' reports under section 432 are published. This threatened to raise BAT's risk to litigation from overseas governments who had lost revenue and encountered difficulties in enforcing public health standards as a result of tobacco smuggling.

According to the sequence of events reported by the Guardian BAT tried to persuade the DTI against a section 432 inquiry. Martin Broughton, the company's chief executive, wrote to Stephen Byers (then Secretary of State for the DTI) twice, asking for an audience, but was rebuffed on both occasions. However, as a member of the multinational chairman's group, a lobbying group composed of leading executives of multinational companies, Broughton was able to press his case directly to the prime minister during a private breakfast at Number Ten. Following the meeting, Byers was summoned to breakfast with the Prime Minister where he was pressured into granting Broughton a formal audience. As *The Guardian* reported at the time, this was despite the fact that the “company stood accused of colluding in cigarette smuggling on an unprecedented scale” [67] and despite the fact that ASH, the antismoking group, had been refused a similar meeting.

At the meeting, Byers agreed to back BAT in its legal fight against the Colombian government, which was bringing a lawsuit in the US over the smuggling allegations. After the meeting, official documents indicate a distinct change in tone in the way in which BAT was discussed within the DTI [67],[138]. BAT was talked about as one of Britain's world-class companies and Byers's civil servants pressed him to cancel the planned inquiry, arguing there was insufficient evidence. Whilst accepting that the Health Committee's intervention necessitated some sort of formal response, Byers was persuaded to launch the investigation under section 447 of the Companies Act, which grants inspectors more limited powers of questioning and which does not lead to the publication of a report. The inquiry dragged on for almost 4 years, during which time no further information was released into the public domain. Byers moved on and, finally, under the new trade secretary, Patricia Hewitt, the DTI announced there was insufficient evidence to take the matter further [67].

By 2000, Martin Broughton (BAT's chair between 1998 and 2004) was describing the relationship between the industry and the DoH as a “Mexican stand-off,” [75] contrasting it unfavourably with the company's relationship with previous Conservative administrations, which had been characterised by close dialogue with the DoH over both product modification strategies [75] and the Voluntary Agreement governing tobacco advertising [76] and health warnings in the UK [77]. More limited access to DoH officials had a number of important impacts. It left BAT effectively powerless to challenge the DoH's refusal to act as the industry's advocate in negotiations over the introduction of the EU's Tobacco Products Directive (2001/37/EC) [78] and had potentially serious ramifications for its ability to manage the impact of EU enlargement on its business in Eastern Europe [70]. Relationship building was considered central to managing this uncertain regulatory environment [79], and it was in this context that reestablishing access in the UK became a key objective of BAT's public affairs strategy.

### CSR as a Tool to Reestablish Political Access: BAT and the UK DoH, a Case Study

#### CSR as a means of initiating access

BAT's attempts to reestablish access with the UK DoH in the late 1990s were restricted by widespread, low levels of trust in the tobacco industry [70]. Consequently, senior BAT executives determined that they first had to explore ways of improving public perception of the company, as a letter from Broughton to the company's end markets in October 1998 explains: “The group's image and reputation as an honest and open organisation have suffered recently, mainly as a result of the litigation in the US. The new British American Tobacco plc needs to regain a reputation of being trustworthy and responsive if we are to …. gain the access and influence that we need externally. Much of this will come from being economically successful. However, an important contribution will also come from our reputation as a good corporate citizen.” [80]

Broughton's remarks reflect a long held assumption of a positive correlation between corporate reputation and access, which was key to realising the company′s policy of encouraging operating companies to pursue a “pro-active programme of regular contact with Government officials, politicians and ministers” [81],[82]. They are also consistent with evidence that projects were partly chosen for inclusion in the company's CSRP based on their capacity to facilitate access [83],[84]. In a corporate responsibility budget drafted in 1998 by a consultant brought in by BAT to help develop its CSR strategy, “identify[ing] and support[ing] projects that [had] high political priority and that would enhance BAT's ability to build ‘platforms for dialogue’ with rule-makers in several countries” was underlined as an important consideration [84]. Moreover, faith in the power of stakeholder dialogue as a means of “facilitating access to hitherto ‘uncooperative’ opinion-formers” was consistently cited as a positive reason for investing in social reporting [85],[86]—a key CSR practice in which companies publish an audit of their social performance.

Whilst BAT's early efforts to restore its reputation centred on building its social reporting capacity [87], this was developed alongside a number of other communications platforms that, as one company planning meeting put it, were designed to “enhance understanding of [the] Corporate Brand at a deeper level … in an aligned consistent manner” [88]. One of the most important of these was the company's Partnership for Change programme (PCP). This covered a number of key areas such as voluntary marketing codes, youth smoking initiatives, accommodation of smokers and nonsmokers, and reduced risk cigarettes (see Box 3) [89]. Originally devised as a response to criticisms levelled at the company by the Health Committee inquiry [90], BAT also used the programme as an organising platform to frame its CSR initiatives in the early 2000s. By emphasising the value to public health of meetings between tobacco companies, government officials, and public health groups in the form of summits and fora the initiative was well designed to generate dialogue with the DoH.

**Box 3. BAT Partnership for Change proposals [99],[139]**

Twenty suggestions for progress

1. Define and ensure responsible marketing

“We believe in our right to provide adult smokers with brand choice and information, alongside our responsibility to ensure that our marketing does not undermine efforts to prevent children from smoking. This means, for example, that:

Tobacco advertising should not contain imagery or messages which appeal to children;
Tobacco marketing should not take place in environments used mainly by people under age;
Tobacco advertising should not be misleading.”

Our proposals:

- Establish a forum where the industry, government, public health groups and consumer advocacy groups can reach consensus on what constitutes responsible marketing of tobacco products.
- Fund independent research to determine whether any specific form of marketing has a particular impact on decisions to smoke by under age teenagers.
- In the light of this research, comprehensively review the voluntary code governing tobacco marketing.

2. Ensure that only adults smoke

Our proposals:

- Set up a summit meeting amongst Government, public health groups, educationalists, tobacco companies, and retailers, to develop a UK action plan on under age smoking.
- Fund independent research into teenage behaviour, including decisions to smoke.
- Set up a teenage action group, where teenagers themselves can develop messages to their peers on how to deal with adult products such as cigarettes and alcohol, and also on how to deal with illegal drugs.
- Mobilise teachers, parents, Government and public health bodies in an integrated communications campaign with effective messages.
- Raise the legal age for tobacco purchase in the UK from 16 to 18, in line with alcohol.
- Involve retailers in developing a tighter and more effective enforcement regime to prevent under age sales.
- Provide more support through schools and retail outlets for the CitizenCard, a youth identity scheme that helps retailers confirm a customer's age.
- Research the formal and informal channels through which under age smokers obtain cigarettes, including the rapidly growing UK “black market.”
- Fund independent research into the best excise strategy to make a major reduction in the UK black market in tobacco products.

3. Ensure that the public are appropriately informed of the risks; Ensure that smokers are informed of the varying levels of risk and are therefore encouraged to smoke fewer cigarettes, smoke lighter cigarettes, and quit smoking sooner. We believe that after decades of public education, people are well aware of the health risks associated with smoking. However we also believe there are steps which smokers could take to reduce their exposure to risk, and that public health messages could address these. Government, public health bodies and tobacco companies could work together on such messages to smokers and innovative ways to deliver them.

Our proposals:

- Fund independent research to determine the extent to which risk may be reduced from low tar cigarettes.
- Discussion amongst tobacco companies, Government, public health groups and the medical profession to develop consumer messages on smoking fewer cigarettes, smoking lighter cigarettes, and quitting smoking sooner.

4. Ensure that the desires of non-smokers to avoid the annoyance of smoke are accommodated.

Our proposals:

- Provide funding to BRE (the Building Research Establishment) to investigate cost-effective devices for reducing environmental tobacco smoke in public spaces.
- Support the AIR (Atmosphere Improves Results) campaign in the UK which provides solutions for the hospitality sector.

5. Ensure that the effort to both research and develop lower risk cigarettes, and the communication of those developments to consumers, be encouraged and supported, unencumbered by opportunistic criticism.

Our proposals:

- A scientific forum to discuss which product changes would be supported by public health groups and might gain consumer acceptability, and how new products might be tested.
- Fund independent research on very low tar cigarettes, to determine how much less tar smokers take.
- Ask the International Standards Organisation (ISO) to examine whether current tar and nicotine machine measurements could be improved.
- Consider ways of informing consumers about innovative products, including informational advertising.

In January 2000, Martin Broughton initiated efforts to reestablish contact with the DoH by writing to Alan Milburn, then Secretary of State for Health. Broughton requested a meeting to discuss five areas relating to tobacco and disease that the company had identified as potentially productive areas for working in partnership with Government and public health groups, which broadly corresponded to its PCP [91]. Despite BAT's offer to work in partnership on these issues, Milburn refused to meet.

Three months later, Broughton followed up a discussion with the prime minister, Tony Blair, at the Multinational Chairman's Group (an informal grouping of the heads of the UK's biggest multinational companies who, historically, have enjoyed privileged access to Downing Street over regular breakfast meetings [67],[92]), with a long and detailed letter outlining the case for lower taxation [93]. In two lines tagged on to the end of the letter, Broughton also proposed that the Government consider cooperating with BAT over developing its PCP [93]. Crucially, whilst the Prime Minister's reply rejected all of Broughton's arguments on taxation, he suggested that DoH officials would be “very happy” to meet with Broughton with a view to developing the PCP [94], underlining the way in which CSR's seemingly anodyne nature can help facilitate access.

The Prime Minister's response was consistent with advice already proffered by Stephen Byers, then secretary of state at the DTI, during a meeting with Broughton and BAT's international government affairs manager. A note of this meeting indicates that BAT were hoping to discuss the possibility of the Government abandoning plans for a UK advertising ban [75]. However, by describing the ban as a “manifesto commitment,” Byers effectively scotched this idea. Despite this, the BAT delegation was able to turn the discussion to another PCP initiative—a forum to develop the basic principles of responsible marketing for socially harmful products and services (such as tobacco and gambling). Documents suggest Byers and his team were prepared to discuss this initiative, even though it would potentially conflict with the government's eventual proposals for an advertising ban. Broughton also used the meeting to broach the issue of access with the DoH. Byers assured him that he would speak with Milburn about possible dialogue if BAT could “come up with a 4 or 5 point agenda on ‘common ground for working together’” [75]. On first inspection, the DTI's intervention might indicate that its officials played a part in shaping BAT's CSRP. However, BAT's response to Byers' suggestion that the company “should work with the DTI to pull an agenda together” [75] was largely a restatement of the core elements of its PCP [95]. This suggests that public officials at the DTI had very little input into the terms upon which the meeting with the DoH was set up. In addition to highlighting how sponsoring departments can help facilitate tobacco companies' access to other departments, this illustrates the value of a well-designed set of CSR related messages in setting the agenda of meetings with public officials (see below).

The combined effect of the DTI's assistance and the Prime Ministers' apparent endorsement of PCP appears to have marked a turning point in BAT's efforts to use CSR initiatives to break down barriers to access. Before receiving the Prime Minister's reply Broughton had accepted an invitation to attend a seminar held annually in the Civil Service National College (now the National School of Government) in Sunningdale, which brought together senior civil servants and business leaders [96]. Although a briefing prepared for this meeting also explored how CSR could be used as a means of facilitating access to other parts of government his primary aim was to make contact with Chris Kelly, Permanent Secretary to the DoH [96],[97]. Following the seminar, Broughton appealed to Kelly for further dialogue, asking how BAT might “engage more constructively with regulators, legislators, public health authorities and the academic community” [98]. To underline that BAT wanted to learn more of the DoH's major concerns about tobacco in order to inform its “thinking about how [the company] might be able to contribute appropriately to positive solutions,” Broughton supplied Kelly with a copy of BAT's PCP [99]; assuring him that this represented “a genuine attempt to offer potential starting points for dialogue, especially in areas where we believe we could “bring something to the table to achieve positive results” [99]. To keep the dialogue alive, Broughton asked for feedback on these initial ideas, and, significantly, enclosed a copy of the Prime Minister's reply to underline that dialogue with BAT on its PCP had his approval.

Broughton's efforts were successful in so far as Kelly directed him to Mohammed Haroon, Branch Head of Cancer Prevention and Substance Misuse at the DoH [100]. Responsibility for taking the matter forward within BAT was delegated to Adrian Payne (BAT's International Scientific Affairs Manager and future head of Corporate, Social and Regulatory Affairs) [101]. Summarising his first meeting with Haroon in a note to BAT executives, Payne indicated that whilst Haroon had questioned how realistic it was for the department to accede to his request for dialogue when the industry was simultaneously suing the Government, he was prepared to listen to what Payne had to offer. Further, the fact that Payne also described another DoH official as expressing considerable interest in what he had to say [102], and the fact that he noted that both officials “picked up on the need to obtain consensus on what might be regarded as ‘safer cigarettes’” is consistent with CSR being effective at developing a constructive agenda for discussion, which constituted a more enabling milieu for decisions favouring industry interests (see below) [102].

#### CSR as an instrument of issue definition and furthering access

BAT employees also used CSR initiatives and themes as a means of issue definition to both optimise the probability of subsequent discussions taking place and frame their content [36]. For example, in Broughton's letter to Kelly described above, Broughton opened by assuring Kelly that the “initial ideas” floated were not “in any sense intended to be ‘prescriptive’,” but rather represented “starting points for dialogue.” However, he then immediately directed Kelly's attention to BAT's 20 specific PCP proposals from which he selected several topics (relating, for example, to youth smoking prevention, “sensible regulation,” potential messages about moderation and research on a “‘safer’ cigarette'”), which Broughton was particularly interested in exploring [99] in future discussions.

That BAT personnel deliberately use CSR initiatives (such as youth smoking prevention) and CSR messages (such as the company's commitment to “sensible regulation” and “safer cigarettes”) to define the issues of meetings with public officials is also suggested in Adrian Payne's note of his first meeting with Mohammed Haroon, which records that, as “prearranged at my suggestion, the theme of the meeting was … risk communication and ‘safer’ cigarettes” [102]. The note further indicates that a key aim of using BAT's CSRP in this way was to “establish a dialogue at a more strategic level than the existing TMA (Tobacco Manufacturers' Association) DoH meetings on specific issues such as additives/ingredients” [102]. Although capable of being interpreted in several ways, these passages are consistent with BAT attempting to use CSR initiatives strategically to influence the policy alternatives under discussion within the DoH. The persistent emphasis on safer cigarettes by BAT officials was commercially significant for the company for several reasons. However, a key motivation was the creation of common ground between health ministries and industry scientists. This was regarded as an important first step in rehabilitating the reputation of industry funded science, which, in addition to being seen as crucial to the ability of BAT's in-house scientists to create new products, was considered essential to giving the company a credible voice in policy discussions on how to reduce the risks associated with tobacco use [103].

Using CSR narratives and initiatives as a means for suggesting an agenda for future discussions also provided a platform for BAT employees to request further dialogue with officials. Payne reported that he had wound up the meeting by “restating [the company's] desire for dialogue over a range of issues” [102]. His note suggests that, although cautious, Haroon was open to the idea, responding that “a step by step approach was the best option” because “time was needed to build trust” [102]. Payne's note goes on to explore how he planned to take the matter forward and suggests that using CSR as a means of continuing dialogue and, ultimately, normalising relations between the DoH and BAT was a key objective. In addition to reiterating an earlier suggestion that the DoH participate in a “risk reduction forum” organised by BAT, Payne indicated he would ask DoH officials for feedback on BAT's Web site; consider suggesting that the Department send an observer to one of the company's training sessions on smoking and health messages; solicit the DoH's advice on how to get these messages across “to those in developing countries that can't read or write”; invite DoH officials to tour the company's research and development facilities; and generally encourage discussion of some of the company's other CSR proposals [102]. Significantly, the note also illustrates BAT's awareness of the need to control the way in which the concepts involved in its CSRP were defined and understood by officials, with Payne floating the idea of making a series of presentations to DoH officials so that they were “fully informed and not dependent on third parties' views” [102].

Additional evidence indicates that BAT has continued to use CSR as a means of issue definition; effectively making old arguments against (nonvoluntary forms of) regulation and governance in a new form. For example, at a meeting of an All Party Parliamentary Group on Corporate Responsibility in 2008 [13] Michael Prideaux (Director of BAT's Corporate and Regulatory Affairs department [CORA]), claimed that, by focusing on reducing smoking rates, the FCTC had effectively rejected harm reduction “as a part of a pragmatic approach to public health” [13]. In this way, CSR was used to reframe BAT's long running efforts to reduce the impact of the treaty on tobacco sales as a constructive and responsible response to the health problems associated with tobacco consumption [104].

Although, as in the above examples, the inherent capacity of CSR to define issues for discussion typically facilitates dialogue around specific CSR initiatives and concerns, it may also have long term effects on the relationship between government and the tobacco industry that expedite influence. To this effect, there is some evidence to suggest that CSR-based access is designed to shift relations from low trust, low frequency access to high trust, high frequency access—something that is broadly recognised as being key to political influence across policy domains [105]–[108]. As Payne put it after his first meeting with Haroon: “If we can get a dialogue going it would be a good opportunity for [Martin Broughton] to get together with Alan Milburn to take an umbrella view of the interaction (how could we progress faster?). If we can't get one going they could meet to focus on why not (i.e., what would we have to do to build trust?). As with many of our stakeholder interactions, trust-building is paramount.” [102].

### CSR and the Proliferation of Access Points

The documents also illustrate the way in which CSR has expanded the number of access points across Government, providing BAT with more opportunities to meet and talk to officials. This is perhaps best exemplified by a note from BAT's International Development Affairs Manager outlining civil service contacts with a CSR brief in British Government Departments in preparation for Broughton's Sunningdale meeting. The document records four government departments (the Department for International Development, the DTI, the Department for the Environment, Transport and the Regions, and the Foreign and Commonwealth Office) either with units devoted to, or with strategic interests, in CSR, in addition to the Performance and Innovation Unit in the Cabinet Office which, at the time, oversaw the CSR agenda [97].

BAT's use of these additional access points to change attitudes within government more broadly is illustrated by a written exchange between Michael Meacher, then the Minister for the Environment, and Broughton. Meacher had written to Broughton as part of the Government's strategy on sustainable development in the UK, asking him for a summary of the action BAT was taking to measure, manage, and report on its environmental impact. Meacher noted that he had particular cause to write to Broughton, given that BAT had scored poorly in a recent survey by Pensions Investment Research Consultants' (PIRC, Environmental Reporting 2000). The significant point to note about the letter is that it focused exclusively on the environment, Meacher requesting detailed responses from Broughton to a range of questions (see Box 4) [109]. Despite this, Broughton's lengthy reply opened by discussing the company's PCP proposals, which he claimed exemplified the company's policy of “actively seek[ing] constructive dialogue on many issues relevant to [the] industry, in the spirit of commitment to corporate social responsibility.” Acknowledging that this was not immediately specific to Meacher's questions, Broughton nonetheless invited Meacher to “discuss any of these matters … within the context of the broad social dimension of sustainable development, and would welcome an opportunity to hear [Meacher's] views.” [110]. In the event, Meacher's reply ignored Broughton's invitation [111]. Nonetheless, the exchange highlights how the fuzzy boundaries and negotiation at the core of CSR can allow companies to exploit alternative channels to getting key strategic messages across to officials.

**Box 4. Meacher's initial questions to Broughton [109]**

- Does your company have an environmental policy and, if so, what is it, is it made public and where?
- Is there a board member with specific responsibility for environmental issues?
- What information does your company publish about environmental performance including compliance with relevant laws and regulations?
- Specifically, does your company measure its impact on the environment in terms of greenhouse gas emissions from energy user, waste emissions, and water use. Is this information made public and how?
- Does your company set quantified targets for improvement in these or other environmental impacts and, if so, what are they, are they made public and how?
- What other environmental initiatives does your company carry out or support?

If you cannot yet respond positively to all or some of these questions, but your company is already planning to take action in these areas, I would be grateful if you could also make this clear.

## Discussion

Before discussing the policy implications of our findings, it is important to note that our account gives an incomplete picture. As we explain below, the ability of CSR to facilitate access and create opportunities for issue definition is likely to be context dependant [112]. Our efforts to interview the policymakers mentioned in BAT's documents were unsuccessful, making it difficult for us to fully explore this context and its effects on what officials may have thought about BAT's proposals. Nevertheless, the data suggest that CSR facilitates access and creates opportunities for issue definition in a number of ways.

First, CSR facilitates access by providing a basis for requesting meetings with officials who are reluctant to talk to the industry—something illustrated by the fact that whilst attempts to discuss other issues (e.g., tax) were dismissed outright, officials were still willing to discuss BAT's PCP. In relation to the DoH, CSR appears to have worked to this effect by: (a) allowing the company to open up a dialogue about the importance of cooperation and consensus; (b) conveying a sense that the company was offering, or was prepared to offer, some support for government efforts to reduce tobacco consumption; and (c) adding a vital moral dimension to the company's argument that some level of cooperation between the company and government was desirable by presenting the company's proposals as genuine and in the interests of public welfare. Indeed, one of the key factors behind the ability of CSR to open channels of communication may relate to its use of accessible images and emotive appeals to widely accepted social and political values [113]–[116]. By linking the company's preferred policies to politically salient values such as harm reduction, child health, and the importance of cooperation between business and government, BAT's employees were able to represent dialogue as both the morally right thing to do and benign and, therefore, as unlikely to compromise government policy on tobacco control.

In terms of issue definition, our case study illustrates how BAT then harnessed the initiatives and narratives associated with its emerging CSRP to shape the agendas of meetings, in effect defining key issues once access had been established. Using CSR as a means of defining salient issues [117] allowed the company to reopen dialogue over specific issues that appeared closed for discussion and to shift the focus of talks onto voluntary alternatives to statutory measures or other topics (e.g., reduced harm cigarettes), which were consistent with the company's immediate and long-term commercial interests.

In this respect, BAT's engagement with the DoH illustrates the way in which CSR offers companies with poor social or environmental records a structured environment of dialogue and engagement, which shifts attention away from both the social costs associated with the business and any past behaviour that might cause doubts about the trustworthiness of the company and the relative merits of granting it access. Furthermore, by creating new access points for such companies, it helps normalise engagement and dialogue—a crucial step in this context to restoring the trust necessary for the tobacco industry to reestablish its political authority [107],[118]–[120]. These risks are magnified by the fact that new access points created around CSR largely exist outside of departments and agencies with experience of dealing with the tobacco industry, which means contact is often with public officials who have little knowledge of the company's core business and past conduct. An important point to understand in this context is tobacco firms' ability to absorb the costs associated with political activity. This means that the depth of industry–government contact is primarily determined by officials' attitudes to and beliefs in the value of meeting with representatives of the industry. By increasing industry contact with government, CSR effectively alters the balance of officials' diet of information about tobacco and the tobacco industry in favour of tobacco companies.

There is evidence that our case study of the UK is not an isolated example. A recent report by Corporate Observatory Europe suggests that BAT has also used CSR practices, such as stakeholder dialogue, to transmit policy positions to EU policy makers [121]. Likewise a 2007 presentation by Ben Stevens, now BAT's Finance Director, indicates that a key aim of stakeholder dialogue is to develop cooperative relationships with policymakers, which represent a more effective platform for influencing tobacco regulation [122].

Despite the importance that corporate actors attach to access, it is no guarantee of either issue definition or policy influence. Since 1997, UK government policy on tobacco control has largely been at variance with industry interests [62]. This is consistent with evidence from the documents which suggests that some DoH officials tried to actively manage BAT's expectations of in person meetings by emphasising that agreements to meet and listen to what the company had to say did not mean that either government policy or policy implementation were open to negotiation [102]. In short, whilst CSR may represent an effective medium of issue definition under the right conditions (see below), documents indicate that this is more difficult for corporations where officials are well informed and following a clear, evidence-based public policy agenda formulated independently of economic interests. DoH replies to BAT letters on the draft EU directive reinforce this interpretation of the documents [123]. Despite BAT's failure to translate access into policy outcomes, and despite the fact that the documents only give company officials' explanations of the course of events, it is nonetheless important to stress that CSR was still used successfully by BAT to secure and extend access within an unreceptive policy environment, where public health advocates have been active in highlighting the risks attendant on industry political activity. It is reasonable to hypothesise that the impact of political CSR is likely to be greater under different political administrations or in countries where policy élites have historically been more accommodating of industry interests and where the health and economic impacts of specific policy alternatives favoured by the industry is not as widely understood.

This observation raises a more general point about the impact of economic and institutional factors on the relative effectiveness of political CSR. Broughton's membership of high-level policy groups (the Multinational Chairman's Group) and élite social networks (Sunningdale)—both of which were important to reopening dialogue with the DoH—indicate that officials' perceptions of tobacco and, for that matter, other companies as an important source of capital investment, employment, foreign revenue, and taxation receipts [124],[125] are likely to be a key factor in determining the effectiveness of political CSR. In our case study, these “access drivers” were offset by DoH officials' reluctance to negotiate with BAT on alternatives to policy implementation; suggesting that trust amongst policy élites in companies' ability to provide reliable information is likely to be a key determinant of the impact of political CSR.

This last point may help to explain why companies from other industrial sectors—specifically food and alcohol—are currently enjoying greater success in influencing public health policy in the UK through the government's Public Health Responsibility Deal [126]. The Deal encompasses five cross-sectoral networks established to drive improvements in public health. As presently constituted, corporations and business organisations outnumber nonbusiness organisations and individuals (academics, nongovernmental organisations, representatives of public institutions) two to one in the food and alcohol networks that are responsible for setting immediate public health objectives in these areas [127],[128]. By devolving policy formation and delivery to companies whose products and marketing practices constitute the key proximate drivers of alcohol- and diet-related ill health and mortality this marks a potentially important shift in public health policy towards coregulation [129]–[133]. The organising principles of the Deal draw heavily on the idea that CSR can be exploited to promote public health. Further, devised when the Conservative Party were in opposition, newspaper reports indicate that the existence of the Deal owes much to the success that large food and drink companies have had in using CSR as a means of both gaining access to senior Conservative Party members and developing an alternative agenda for public health policy, which attempts to reconcile public health with business competitiveness [129]. Our findings—and the absence of strong evidence suggesting that coregulation is capable of aligning the business models of big food and drinks companies with the demands of public health [126]—suggest that the role of CSR in the Deal needs to be subjected to closer scrutiny.

Finally, in highlighting the political dimensions of CSR, this paper underlines the importance of parties to the FCTC acting on the Guidelines for implementation of Article 5.3 [134]. Article 5.3 was specifically introduced to protect health policies from tobacco industry influence [135]. Its impact depends on governments implementing the Guidelines that comprise a number of Guiding Principles and Recommendations [135]. Recommendations 6.1 and Guiding Principles 2 and 3 are particularly relevant to our findings (see Box 5) [134],[136]. Recommendation 6.1, one of four recommendations that relate to political CSR, states that parties should ensure that all branches of government and the public are informed and made aware of the true purpose and scope of activities described as socially responsible performed by the tobacco industry. Guiding Principles 2 and 3 emphasise the importance of interactions between the tobacco industry and government being transparent (principle 2) and that parties to the Convention require the tobacco industry to provide government officials with information that facilitates the effective monitoring of tobacco industry political activity (principle 3). Although the events described in this paper predate the FCTC, they highlight the importance of ensuring that public officials in nonhealth government departments (such as the DTI, now the Department of Business, Innovation and Skills) are appropriately briefed on the underlying political motivations of tobacco industry CSR and given advice on how to respond to the industry in light of the general intent of Article 5.3. Second, they underline the importance of all meetings with representatives from the tobacco industry being formally minuted and made publicly available either through government Web sites or through freedom of information legislation. This is presently not the case in the UK in relation to meetings of the Multinational Chairman's Group, which was the subject of a recent ruling by the UK Information Commissioner. The Commissioner ruled that minutes and correspondence of the meetings are not disclosable under the Freedom of Information Act on the basis that they relate to the formulation and development of government policy and are, therefore, exempt under section 35(1)(a) of the Act. In light of the findings of this paper, this decision needs to be revised in relation to tobacco companies to bring it into line with the Guidelines for Implementation of Article 5.3.

**Box 5. Recommendation 6.1 and Guiding Principles 2 and 3 of Article 5.3 of the FCTC [134]**

Recommendation 6.1: Parties should ensure that all branches of government and the public are informed and made aware of the true purpose and scope of activities described as socially responsible performed by the tobacco industry.

Guiding Principle 2: Parties, when dealing with the tobacco industry or those working to further its interests, should be accountable and transparent.

Guiding Principle 3: Parties should require the tobacco industry and those working to further its interests to operate and act in a manner that is accountable and transparent.

## Abbreviations

BAT: British American Tobacco
CSR: corporate social responsibility
CSRP: corporate social responsibility programme
DoH: Department of Health
DTI: Department of Trade and Industry
EU: European Union
FCTC: Framework Convention on Tobacco Control
PCP: Partnership for Change Programme
